# Factors and Molecular Mechanisms Influencing the Protein Synthesis, Degradation and Membrane Trafficking of ASIC1a

**DOI:** 10.3389/fcell.2020.596304

**Published:** 2020-10-23

**Authors:** Yayun Xu, Feihu Chen

**Affiliations:** ^1^Department of Epidemiology and Biostatistics, School of Public Health, Anhui Medical University, Hefei, China; ^2^The Key Laboratory of Major Autoimmune Diseases of Anhui Province, Anhui Institute of Innovative Drugs, School of Pharmacy, Anhui Medical University, Hefei, China; ^3^The Key Laboratory of Anti-inflammatory and Immune Medicines, Ministry of Education, Hefei, China

**Keywords:** factors, membrane trafficking, molecular mechanisms, protein expression, acid-sensing ion channel 1a

## Abstract

Acid-sensing ion channels (ASICs) are members of the degenerin/epithelial sodium channel superfamily. They are extracellular pH sensors that are activated by protons. Among all ASICs, ASIC1a is one of the most intensively studied isoforms because of its unique ability to be permeable to Ca^2+^. In addition, it is considered to contribute to various pathophysiological conditions. As a membrane proton receptor, the number of ASIC1a present on the cell surface determines its physiological and pathological functions, and this number partially depends on protein synthesis, degradation, and membrane trafficking processes. Recently, several studies have shown that various factors affect these processes. Therefore, this review elucidated the major factors and underlying molecular mechanisms affecting ASIC1a protein expression and membrane trafficking.

## Introduction

As a member of the degenerin/epithelial sodium channel (DEG/ENaC) superfamily, acid-sensing ion channel 1a (ASIC1a) senses pH changes and has an extensive distribution pattern and function in the peripheral tissues and central nervous system (Radu et al., 2016). ASIC1a is sensitive to extracellular acidification and is involved in several acidosis-related pathophysiological processes, including inflammation (Duan et al., 2007), hypoxia (Tan et al., 2011), and pain (Deval et al., 2010). Plasma membrane expression is critical to the function of ASICs that act as extracellular proton sensors (Waldmann and Lazdunski, 1998). Protein synthesis and degradation, as well as dynamic trafficking processes partially determine the number and function of receptors present on the plasma membrane (Zeng et al., 2014). Therefore, elucidating the factors and the underlying molecular mechanisms that affect ASIC1a expression and membrane trafficking will improve our understanding of its pathophysiological functions in multiple diseases.

## Factors and Molecular Mechanisms Influencing Protein Synthesis, Degradation, and Membrane Trafficking of ASIC1a

Several studies have demonstrated that protein synthesis, degradation, and membrane trafficking of ASIC1a are affected by various factors. Since the number of ASIC1a present on the cell surface correlates with its total expression (Wu et al., 2016), the presence of the channel in the cytoplasm can be used as a pool for supplying to the membrane. Therefore, it is of great significance to explore the factors that influence both the total expression and membrane trafficking of ASIC1a. These factors are highlighted in Table 1.

**TABLE 1:** The factors that regulate the total expression and membrane trafficking of ASIC1a.

Factors	Total expression	Membrane trafficking	References
Acidosis	↑		Dai et al., 2017; Gao et al., 2019; Zu et al., 2020
Hypoxia	↑		Tan et al., 2011; Jernigan et al., 2012
**Inflammatory cytokines**			
IL-6	↑		Zhou et al., 2015
IL-18	↑		Zhou et al., 2018
TNF-α	↑		Zhou et al., 2018
**Neurotrophins**			
NGF	↑		Matricon et al., 2013; Wei et al., 2020
PDGF	↑	↑	Zuo et al., 2019
BDNF		↑	Duan et al., 2012
**Hormones**			
β-estradiol	↓		Zhou et al., 2019a; Song et al., 2020
Insulin	↓		Chai et al., 2010
**Drugs**			
Ibuprofen	↓		Sun et al., 2014
Aspirin	↓		Wu et al., 2019
Omeprazole	↑		Thongon et al., 2014
Amiloride	↓		Sun et al., 2014
PcTx1	↓	↓	Duan et al., 2012; Gao et al., 2019; Wu et al., 2019
Ginsenoside-Rd	↓		Zhang et al., 2012
**MicroRNAs**			
miR-149	↓		Jiang and Zha, 2017
miR-144	↓		Jiang and Zha, 2017
let-7	↑		Jiang and Zha, 2017
**Effector proteins**			
p11		↑	Donier et al., 2005
PICK1		↑	Jin et al., 2010
Rho		↑	Herbert et al., 2018
SGK-1		↓	Arteaga et al., 2008
AP2		↓	Zeng et al., 2013
Dynamin1		↓	Zeng et al., 2013
**Chemicals**			
Acute ethanol	↓		Zhou et al., 2019b
H_2_O_2_		↓	Zha et al., 2009
NO	PFC↑, hippocampus↓		Li et al., 2019
*N*-glycosylation		↑	Jing et al., 2012

**FIGURE 1:**
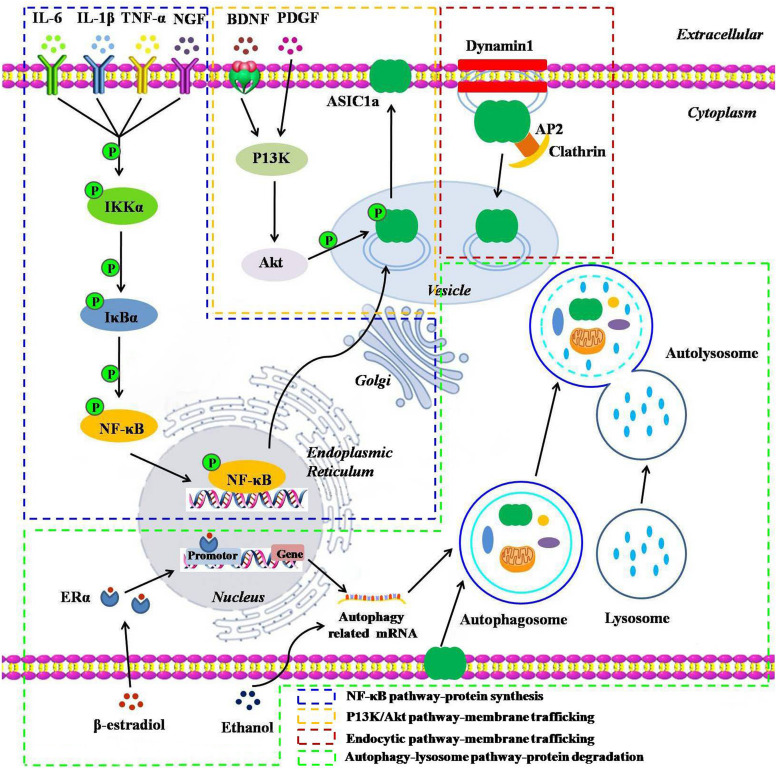
A schematic representation of the underlying molecular mechanisms influencing the protein synthesis, degradation, and dynamic trafficking processes of ASIC1a. The NF-κB pathway: inflammatory cytokines including IL-6, IL-1β, TNF-α, and NGF and their receptor interactions activate the NF-κB pathways, leading to the translocation of NF-κB p65 into the nucleus and enhancing the ASIC1a gene promoter activity, thereby upregulating ASIC1a expression; The PI3K/Akt pathway: BDNF/TrkB or PDGF activates the intracellular PI3K/Akt pathway, and then, induces ASIC1a phosphorylation in vesicle and forward targeting to the plasma membrane; The endocytic pathway: downregulation of ASIC1a surface expression in a clathrin- and dynamin-dependent endocytosis; Autophagy-lysosome pathway: β-estradiol/ERα or acute ethanol exposure enhances ASIC1a protein degradation via the autophagy-lysosome pathway.

The number of receptors present on the plasma membrane partly depends on protein synthesis, degradation, and dynamic trafficking processes. Among them, dynamic trafficking processes mainly include sorting and forward trafficking of the surface receptors from the endoplasmic reticulum through the Golgi apparatus to the plasma membrane via endocytosis and exocytosis (Zeng et al., 2014). An in-depth understanding of the mechanisms that affect the cell surface expression of ASIC1a is essential for a better understanding of cell signal transduction under acidic conditions (Yang et al., 2012). Therefore, we have provided the details of the underlying molecular mechanisms that govern ASIC1a protein synthesis, degradation, and dynamic trafficking (Figure 1).

### Acidosis and Hypoxia

ASIC1a has been characterized as a potent proton sensor for detecting extracellular acidification in the peripheral tissues and brain (Cheng et al., 2018). Our previous studies have indicated that extracellular acidosis increases protein expression of ASIC1a in a pH- and time-dependent manner in rat articular chondrocytes (Dai et al., 2017; Gao et al., 2019; Zu et al., 2020). Moreover, it has been reported that ASIC1a expression is significantly increased in the articular cartilage of adjuvant arthritis (AA) rats (Zhou et al., 2015), the articular synovial fluid of which showed a low pH value compared to that of the non-arthritic rat synovial fluid (Zhou et al., 2018). Furthermore, blocking ASIC1a expression through pretreatment with the ASIC1a-specific blocker such as psalmotoxinf (PcTx1) or ASIC1a RNA interference (RNAi) reversed the promoting effect of extracellular acidification on the protein and mRNA expression levels of ASIC1a and decreased cell death induced by extracellular acidosis (Gao et al., 2019). Similarly, after PcTx1 treatment, both the severity of disease in AA rats and the protein expression of ASIC1a in the synovial tissue decreased *in vivo* (Qian et al., 2020). These results suggest that the injury caused by extracellular acidification might be related to the upregulation of ASIC1a expression.

Hypoxia triggers pathological processes by producing an acidic microenvironment in multiple diseases (Damgaci et al., 2018). Additionally, an increasing number of studies suggest that pathological hypoxia can alter the activity of ASICs. This might provide a progressive understanding of the hypoxic effects in cancer, arthritis, and ischemic brain injury (Yingjun and Xun, 2013). The expression and function of ASIC1a were upregulated after hypoxia in cultured retinal ganglion cells, and PcTx1 was able to reduce cell death *in vitro* (Tan et al., 2011). These results indicate that ASIC1a activation plays a role in cell death induced by hypoxia. Additionally, exposure to chronic hypoxia upregulated ASIC1 protein expression in pulmonary arterial smooth muscle. Furthermore, PcTx1 prevented enhanced store-operated Ca^2+^ entry in pulmonary vascular smooth muscle (Jernigan et al., 2012), suggesting that upregulation of ASIC1 expression might play a role in vasoconstriction during pulmonary hypertension.

### Inflammatory Cytokines

Our recent studies have concentrated on determining the relationship between inflammatory cytokines and ASIC1a expression in rheumatoid arthritis (RA). The results showed that several pro-inflammatory cytokines, including interleukin-6 (IL-6) (Zhou et al., 2015), IL-1β (Zhou et al., 2018), and tumor necrosis factor-α (TNF-α) (Zhou et al., 2018) upregulate the levels of ASIC1a in a time- and dose-dependent manner in articular chondrocytes. Moreover, this effect was partially reversed by pretreating the cells with pyrrolidine dithiocarbamate (PDTC), an inhibitor of nuclear factor kappa B (NF-κB), indicating that pro-inflammatory cytokines induced the upregulation of ASIC1a in articular chondrocytes mainly through the NF-κB signaling pathway.

NF-κB is an evolutionarily conserved transcription factor involved in the expression of genes that play a critical role in various biological processes, including immune response, inflammation, proliferation, and apoptosis (Mitchell and Carmody, 2018). For instance, IL-1β and TNF-α play a role in ASIC1a protein synthesis through the NF-κB signaling pathway in the following ways (Zhou et al., 2018): (1) Co-expression of ASIC1a and NF-κB p65 was high in articular cartilage tissues, especially in AA rats, an experimental animal model of RA; (2) IL-1β or TNF-α increased the translocation of NF-κB p65 to the nucleus in a time-dependent manner; (3) IL-1β- or TNF-α-induced ASIC1a expression was partially abrogated by PDTC; (4) IL-1β or TNF-α enhanced the activity of the ASIC1a gene promoter by increasing the DNA-binding activities of NF-κB, which could be inhibited by PDTC. These results demonstrate that NF-κB activation is involved in the synthesis of the ASIC1a protein induced by IL-1β or TNF-α.

### Neurotrophins

Nerve growth factor (NGF), which regulates cell development and proliferation, has recently been identified as a mediator of the inflammatory response (Rocco et al., 2018). Similar to the regulatory effect of the pro-inflammatory cytokines on ASIC1a expression, NGF was also observed to increase mRNA and protein expression of ASIC1a in a dose- and time-dependent manner in chondrocytes (Wei et al., 2020). The NF-κB signaling pathway was also found to be involved in NGF governing the expression of ASIC1a (Wei et al., 2020).

Brain-derived neurotrophic factor (BDNF) and its neurotrophin receptor, TrkB, play important roles in neuronal plasticity and in the pathophysiology of various brain disorders. It has been recently proposed that BDNF/TrkB signaling is involved in regulation of the ASIC1a membrane trafficking process (Duan et al., 2012). Activation of phosphoinositide 3-kinase (PI3K) has been reported to promote the membrane trafficking of voltage-dependent Ca^2+^ channels (Viard et al., 2004). Moreover, activation of the PI3K/Akt signaling pathway promotes both the total expression and the number of ENaCs present in the membrane (Qi et al., 2014). The PI3K/Akt signaling pathway is also involved in the membrane trafficking process of ASIC1a, similar to its effect on ENaCs. Specifically, activation of TrkB by BDNF stimulated the intracellular PI3K-protein kinase B/Akt pathway, induced ASIC1a phosphorylation, and targeted the neuronal surface in both rat spinal dorsal horn neurons and heterologous cell cultures. However, the co-administration of PI3K and Akt inhibitors attenuated the process, indicating a critical role of the PI3K/Akt pathway in BDNF-mediated upregulation of ASIC1a membrane trafficking. Further research confirmed the stimulatory effect of BDNF on the cell surface expression of ASIC1a, which was eliminated by mutation of the ASIC1a cytoplasmic residue Ser-25 (Duan et al., 2012), suggesting that Ser-25 is a functionally relevant phosphorylation site.

A similar signaling pathway is also involved in the process of platelet-derived growth factor (PDGF), regulating the expression and membrane trafficking of ASIC1a (Zuo et al., 2019). The expression and membrane trafficking of ASIC1a protein were remarkably increased in PDGF-stimulated hepatic stellate cells. However, these effects could be prevented by inhibiting activation of the PI3K/Akt signaling pathway with the inhibitor LY294002 (Zuo et al., 2019), revealing that PDGF stimulated the expression and membrane trafficking of ASIC1a via the PI3K/Akt pathway.

### Hormones

Experimental and clinical data support a pathogenic role of estrogen metabolism and deficiency in RA (Islander et al., 2011; Dupuis et al., 2018). Our recent study demonstrated that ASIC1a is involved in the mechanism underlying estrogen replacement therapy in RA. It has been shown that the viability of chondrocytes was improved by pretreating the cells with PcTx1, an inhibitor of ASIC1a. Similar to the effect of PcTx1, pretreatment with β-estradiol also improved cell viability. The combined effect of β-estradiol and PcTx1 on the cells did not show an additive effect. Moreover, the effect was similar to that observed when PcTx1 was administered alone, suggesting that inhibiting ASIC1a is likely to involve β-estradiol-mediated protection. Furthermore, β-estradiol was able to downregulate the expression of ASIC1a protein through estrogen receptor α (ERα) and protect the chondrocytes from acid-induced damage and apoptosis (Song et al., 2020). Further studies indicate that downregulation of ASIC1a protein expression can be attributed to β-estradiol, which promotes the degradation of ASIC1a protein through the autophagy-lysosomal pathway (Song et al., 2020). These findings suggest that β-estradiol has the potential to be developed as a novel strategy for the treatment of RA by downregulating ASIC1a protein expression. Activation of ASIC1a induced by tissue acidification, a salient feature of cerebral ischemia, plays a vital role in the progression of ischemia (Xiong et al., 2004; Li et al., 2016a). Recent *in vitro* and *in vivo* studies suggested that β-estradiol can protect neurons against the acidosis-mediated neurotoxicity and ischemic brain injury, possibly by suppressing ASIC1a protein expression (Zhou et al., 2019a). β-estradiol reduced the protein expression of ASIC1a in cerebral ischemia by promoting protein degradation through ERα, similar to its effect on ASIC1a protein expression in RA (Zhou et al., 2019a). These results highlight a novel mechanism underlying the protective effect of β-estradiol in tissue acidification-related diseases.

Insulin participates in the neuronal function by modulating expression of the various ion channels and neurotransmitter receptors on the cell surface (Wan et al., 1997; Skeberdis et al., 2001). Additionally, insulin was recently identified as a regulator of the ASIC1a membrane trafficking (Chai et al., 2010) and can maintain a low level of ASIC1a on the plasma membrane. In contrast, intracellular ASIC1a was transported to the cell surface during insulin deficiency, leading to an increase in ASIC1a expression on the membrane (Chai et al., 2010).

### Drugs

Non-steroidal anti-inflammatory drugs (NSAIDs) are used to treat the inflammation- and pain-related disorders. Accumulating evidence links certain aspects of NSAID pharmacology with ASICs (Voilley et al., 2001; Sun et al., 2014). ASIC1 expression was upregulated in the nucleus pulposus cells due to chronic degeneration induced by the acidic conditions, and this effect was attenuated by treating the cells with ibuprofen, a widely used NSAID (Sun et al., 2014). Similarly, aspirin, another classic NSAID, was reported to inhibit the upregulation of ASIC1a mRNA and protein expression in the chondrocytes of AA rats (Wu et al., 2019).

Amiloride, a non-specific blocker of ASICs, is used to study the functions of ASICs (Leng and Xiong, 2013). Our previous study demonstrated that protein expression of ASIC1a was upregulated in the chondrocytes of AA rats, which was reversed by treating the cells with amiloride (Wu et al., 2019). Another study revealed that the increased expression of ASIC1 in the nucleus pulposus cells of the human intervertebral disc during degeneration was inhibited by amiloride treatment at a concentration of 1 mmol/L (Sun et al., 2014). Further research is needed to explore the effect of amiloride on the expression of ASIC1a at lower concentrations, since 1 mmol/L is an extremely high concentration for *in vitro* experiments.

PcTx1, the peptide toxin obtained from spiders, is a gating modifier of ASIC1a and has been widely used to explore the functions of ASIC1a (Leng and Xiong, 2013). Our previous studies have confirmed that PcTx1 reversed the enhancing effect of extracellular acidification on ASIC1a protein and mRNA expression in articular chondrocytes (Gao et al., 2019; Wu et al., 2019). Similarly, the increased expression and membrane trafficking of ASIC1a induced by PDGF stimulation were inhibited by PcTx1 (Zuo et al., 2019).

Omeprazole, a well-known proton-pump inhibitor, is frequently prescribed for the treatment of peptic ulcers through the anti-gastric mechanism mediated by acid secretion. Thongon et al. (2014) recently demonstrated that omeprazole enhanced ASIC1a expression in Caco-2 cells, leading to the inhibition of paracellular Mg^2+^ absorption via a Ca^2+^-dependent pathway.

Ginsenoside (GS)-Rd, the major active compound present in *Panax ginseng*, has neuroprotective effects against ischemic stroke (Nabavi et al., 2015; Zhang et al., 2016). A possible link between the neuroprotective effect of GS-Rd and ASIC1a activity has been hypothesized, since the pH of brain tissue can usually drop below 6.0 during severe ischemia (Wang et al., 2015). The rat middle cerebral artery occlusion (MCAO) model was used to investigate the effects of GS-Rd on the expression of ASICs in ischemic stroke. The results showed that the mRNA and protein expression levels of ASIC1a and ASIC2a were remarkably increased after stimulation with GS-Rd in the MCAO model. Pretreatment with GS-Rd not only attenuated ASIC1a upregulation, but also promoted ASIC2a expression (Zhang et al., 2012). These results indicate the neuroprotective effects of GS-Rd following cerebral ischemia may be related to its differential regulation in ASIC1a and ASIC2a expression.

### MicroRNAs

Over the past decade, microRNA has emerged as an important group of regulatory molecules in controlling ion channels (Gross and Tiwari, 2018). A recent study by Zha et al. explored the effects of several miRNAs on the expression of ASIC1a, and they found that miR-144 and -149 reduced ASIC1a expression while let-7 increased ASIC1a protein expression (Jiang and Zha, 2017). In a subsequent study, it was confirmed that miR-149 targets the 3′-untranslated region of ASIC1a to regulate protein expression (Jiang and Zha, 2017). Collectively, further studies may provide an alternative to manipulate the expression of ASIC1a in acidosis-related diseases.

### Effector Proteins

Annexin II light chain p11, a member of the S100 family of small and dimeric EF-hand Ca^2+^-binding proteins, has been demonstrated to control the number of ENaCs present in the membrane via the exocytic pathway (Cheung et al., 2019). Considering that ASICs and ENaCs are both members of the DEG/ENaC gene family (Boscardin et al., 2016), it is rational to assume that p11 may have a regulatory effect on ASICs. Therefore, it was not surprising that p11 physically interacted with the N-terminus of ASIC1a (Donier et al., 2005). Moreover, an interaction between p11 and ASIC1a was demonstrated by immunoprecipitation in rat dorsal root ganglion *in vivo* (Donier et al., 2005). Furthermore, the co-expression of p11 and ASIC1a in CHO-K1 cells led to a twofold increase in ASIC1a expression on the plasma membrane (Donier et al., 2005). These results indicated that p11 might have a role in regulating ASIC1a expression on the plasma membrane. Additionally, further research is required to confirm whether the underlying mechanism by which p11 regulates ASIC1a expression on the plasma membrane is similar to that of regulating ENaC expression through the exocytic pathway.

Protein interacting with C kinase 1 (PICK1) is a peripheral membrane protein that regulates trafficking of diverse membrane proteins (Li et al., 2016b). Several studies have indicated a close connection between PICK1 and ASIC1a. First, ASIC1a has been shown to interact with the PDZ domain of PICK1 through its C-terminus, and this interaction changes the subcellular distribution of ASIC1a (Duggan et al., 2002; Hruska-Hageman et al., 2002). Second, PICK1 overexpression increases the expression of ASIC1a on the cell surface, which depends on the BAR domain of PICK1 (Jin et al., 2010). Third, knockout of the gene *PICK1* is attributed to the decreased expression of ASIC1a and ASIC2a proteins on the plasma membrane (Hu et al., 2010). Fourth, the link between PICK1 and ASIC1a is regulated by protein kinases, including protein kinase (PK) A and PKC (Leonard et al., 2003; Hu et al., 2010). These findings provide compelling evidence that blocking the link between ASIC1a and PICK1 can be used as a treatment for ASIC1a-mediated diseases.

RhoA, a small G protein of the Rho family, has been demonstrated to promote ENaC trafficking to the plasma membrane, thereby increasing its activity (Staruschenko et al., 2004; Pochynyuk et al., 2007). Similarly, activation of RhoA increased ASIC1a expression on the plasma membrane and enhanced store-operated Ca^2+^ entry in the pulmonary arterial smooth muscle cells (Herbert et al., 2018), which might help improve our understanding of the vital role of RhoA in the pathogenesis of pulmonary hypertension.

Serum- and glucocorticoid-inducedkinase-1 (SGK1) plays an important role in the modulation of ion channels and regulation of ENaCs (Lang and Pearce, 2016). Recently, SGK1.1, a spliced isoform of SGK1, has been found to downregulate the activity of neuronal ASIC1, at least in part, by decreasing the expression of the channels on the plasma membrane (Arteaga et al., 2008).

There is extensive amount of evidence that suggests both ENaCs and ASICs are regulated by clathrin-dependent endocytosis (Wang et al., 2006; Zeng et al., 2014). Clathrin adapter protein 2 (AP2), a heterotetrameric complex containing αβ_2_μ_2_σ_2_ subunits, links membrane proteins to clathrin, which initiates clathrin assembly at the cell surface (McMahon and Boucrot, 2011). Tyrphostin A23, a pharmacological clathrin-mediated endocytosis inhibitor, reduced the association of clathrin with AP2 in the membrane (Wang et al., 2016), and increased the expression level of ASIC1a on the membrane in both mouse cortical neurons and heterologous cells, indicating a regulative role of clathrin-mediated endocytosis in the surface density of ASIC1a (Zeng et al., 2013). Moreover, knockdown of AP2μ2 (a core subunit of the AP2 complex) also enhanced the surface density of ASIC1a, indicating a critical role of the AP2 complex in ASIC1a internalization.

Dynamin is a large GTP are responsible for diverse cellular processes, including endocytosis, and plays a crucial role in vesicle scission after cargo internalization (Antonny et al., 2016). Blocking the constitutive endocytosis of ASIC1a with the dominant-negative dynamin1 K44A or dynasore, a small-molecule dynamin inhibitor, increased the surface density of ASIC1a protein (Zeng et al., 2013). These results indicate that ASIC1a undergoes constitutive clathrin- and dynamin-dependent endocytosis, resulting in downregulation of ASIC1a expression at the cell surface.

### Chemicals

It is now widely known that acute ethanol administration has neuroprotective effects during cerebral ischemia (Wang et al., 2012). Tissue acidification and its associated activation of ASIC1a are common features of cerebral ischemia (Xiong et al., 2004). Our recent study showed that the neuroprotective effect of ethanol might be related to the regulation of ASIC1a expression in neurons against acidosis-induced neurotoxicity (Zhou et al., 2019b). It has been indicated that acute treatment of neurons with ethanol decreased ASIC1a protein expression and acid-induced [Ca^2+^] elevation (Zhou et al., 2019b). Further evidence suggests that the downregulation of ASIC1a protein was mediated by degradation of the protein via the autophagy-lysosome pathway (Zhou et al., 2019b).

Hydrogen peroxide (H_2_O_2_) is an endogenous reactive oxygen species that contributes to oxidative stress (Murphy and Friedman, 2019). A previous study showed that the application of oxidants inhibited ASIC1a currents in cultured mouse cortical neurons (Chu et al., 2006). More importantly, H_2_O_2_ has been reported to affect the links between the three subunits of ASIC1a (Zha et al., 2009). Previous studies have demonstrated that inter-subunit disulfide bonds could form intracellularly between ion channel subunits, including ASIC1a. Inter-subunit disulfide bonds can produce ASIC1a complexes that are larger than the trimers (Zha et al., 2009). Given that ASIC1a presents on the cell membrane as a trimer, these cytoplasmic ASIC1a complexes that are larger than the trimers combine by disulfide bonds to affect the transport of ASIC1a to the cell membrane, leading to a decrease in their cell surface expression. By targeting the C-terminal cysteines, H_2_O_2_ increases the inter-subunit disulfide bond formation, leading to the reduced expression of ASIC1a located on the cell membrane and reduced H^+^-gated current (Zha et al., 2009).

Nitric oxide (NO) is a free radical signaling molecule that regulates numerous physiological and pathological conditions. NO signaling has been shown to increase the expression of transient receptor potential vanilloid type 2, a calcium channel, and its trafficking to the plasma membrane via a PI3K dependent pathway (Maksoud et al., 2019), suggesting that NO signaling has the potential to regulate the expression of membrane proteins. Recently, differential regulatory effects of NO signaling on ASIC1a expression in the prefrontal cortex (PFC) and hippocampus have been confirmed. Microinjection of *S*-nitroso-*N*-acetyl-D, L-penicillamine, an NO donor, upregulated the expression of ASIC1a in the PFC and downregulated its expression in the hippocampus. In contrast, 7-nitroindazole, an nNOS inhibitor, showed the opposite effect on the regulation of ASIC1a expression in the PFC and hippocampus (Li et al., 2019).

### *N*-Glycosylation

*N*-glycosylation, a ubiquitous protein modification, alters the molecular and functional features of glycoproteins and is involved in various physiological processes and diseases. A large number of studies have demonstrated that *N*-glycosylation of the extracellular domains of some membrane proteins is important for maturation and apical location of these proteins (Vagin et al., 2009; Moremen et al., 2012). A close relationship between *N*-glycosylation and ASIC1a membrane expression has been observed in several studies: (1) there was a high proportion of glycosylated ASIC1a on the surface of CHO cells and hippocampal neurons, indicating that mature ASIC1a was preferentially transported to the cell surface (Jing et al., 2012); (2) inhibition of glycosylation with tunicamycin reduced ASIC1a surface transport (Jing et al., 2012); (3) disrupting the interaction between the first transmembrane domain and the thumb of ASIC1a altered ASIC1a folding, inhibited its glycosylation, and reduced its surface trafficking (Jing et al., 2011).

## Conclusion

Recent studies have demonstrated that ASIC1a plays a crucial role in the occurrence and development of diseases related to the central and peripheral nervous system, and it is considered to be a potential therapeutic target. Elucidating the factors and the underlying molecular mechanisms affecting ASIC1a protein expression and membrane trafficking is necessary to better understand the role of ASIC1a in various pathophysiological conditions. This review focused on these topics and summarized the currently known factors that affect ASIC1a expression and membrane transfer and the possible underlying mechanism of ASIC1a synthesis and degradation, as well as membrane trafficking. Several studies suggest that ASIC1a is a promising therapeutic target in acidosis-related diseases.

In order to explore whether ASIC1a can be a potential therapeutic target, several questions remain to be answered. These questions are related to (1) the dynamic changes in ASIC1a expression and membrane transfer during disease progression; (2) the contribution of ASIC1a synthesis, membrane transfer, and degradation in the pathogenesis of diseases; (3) the identification of novel drugs that specifically block ASIC1a and have fewer side effects than the conventional drugs (for example, amiloride can be structurally modified to improve the specific blocking of ASIC1a); and (4) isoform-specific membrane trafficking motifs and the related accessory proteins of ASIC1a. All of these require further confirmation.

In conclusion, although multiple lines of evidence suggest that ASIC1a is an important contributor to multiple acidosis-related diseases and various factors affect its expression and membrane transfer, additional clinical studies are needed to confirm its therapeutic efficacy and safety.

## Author Contributions

YX and FC designed this work and revised the manuscript. YX wrote the manuscript. Both authors contributed to the article and approved the submitted version.

## Conflict of Interest

The authors declare that the research was conducted in the absence of any commercial or financial relationships that could be construed as a potential conflict of interest.
